# Harmonized procedure coding system for surgical procedures and analysis of surgical site infections (SSI) of five European countries

**DOI:** 10.1186/s12874-022-01702-w

**Published:** 2022-08-12

**Authors:** Sibylle C. Mellinghoff, Caroline Bruns, Rouvier Al-Monajjed, Florian B. Cornely, Maria Grosheva, Jürgen A. Hampl, Carolin Jakob, Felix C. Koehler, Max Lechmann, Bijan Maged, Christina Otto-Lambertz, Robert Rongisch, Jule Rutz, Jon Salmanton-Garcia, Georg Schlachtenberger, Jannik Stemler, Janne Vehreschild, Sophia Wülfing, Oliver A. Cornely, Blasius J. Liss

**Affiliations:** 1 Department I for Internal Medicine, Excellence Centre for Medical Mycology (ECMM), University Hospital Cologne, Kerpener Str. 62, 50937 Cologne, Germany; 2 Cologne Cluster of Excellence in Cellular Stress Responses in Aging-Associated Disease (CECAD), University of Cologne, Cologne, Germany; 3 German Centre for Infection Research (DZIF), Partner Site Bonn-Cologne, Cologne, Germany; 4 Department of Urology, University Hospital Düsseldorf, Düsseldorf, Germany; 5 Department of Otorhinolaryngology, Head and Neck Surgery, University of Cologne, Cologne, Germany; 6 Center of Neurosurgery, Department of General Neurosurgery, University Hospital Cologne, Cologne, Germany; 7 Department II of Internal Medicine and Centre for Molecular Medicine Cologne, University of Cologne, Faculty of Medicine and University Hospital Cologne, Cologne, Germany; 8 Department of Trauma Surgery, Orthopaedic Surgery and Sports Traumatology, Witten/Herdecke University, Sana Medical Centre Cologne, Cologne, Germany; 9 Department of Orthopedics and Trauma, Surgery University Hospital of Cologne, Cologne, Germany; 10 Department of Dermatology and Venereology, University of Cologne, Faculty of Medicine and University Hospital Cologne, Cologne, Germany; 11 Department of Thoracic and Cardiovascular Surgery, University Hospital of Cologne, Cologne, Germany; 12 Department of Internal Medicine, Haematology/Oncology, Goethe University Frankfurt, Frankfurt, Germany; 13 Department of Gynecology, Vivantes Klinikum Neukölln, Berlin, Germany; 14 Clinical Trials Centre Cologne (ZKS Köln), Cologne, Germany; 15 Department I of Internal Medicine, Helios University Hospital Wuppertal, Wuppertal, Germany; 16 School of Medicine, Faculty of Health, Witten/Herdecke University, Witten, Germany

**Keywords:** International procedure code, Surgical procedure

## Abstract

**Background:**

The use of routine data will be essential in future healthcare research. Therefore, harmonizing procedure codes is a first step to facilitate this approach as international research endeavour. An example for the use of routine data on a large scope is the investigation of surgical site infections (SSI). Ongoing surveillance programs evaluate the incidence of SSI on a national or regional basis in a limited number of procedures. For example, analyses by the European Centre for Disease Prevention (ECDC) nine procedures and provides a mapping table for two coding systems (ICD9, National Healthcare Safety Network [NHSN]). However, indicator procedures do not reliably depict overall SSI epidemiology. Thus, a broader analysis of all surgical procedures is desirable. The need for manual translation of country specific procedures codes, however, impedes the use of routine data for such an analysis on an international level. This project aimed to create an international surgical procedure coding systems allowing for automatic translation and categorization of procedures documented in country-specific codes.

**Methods:**

We included the existing surgical procedure coding systems of five European countries (France, Germany, Italy, Spain, and the United Kingdom [UK]). In an iterative process, country specific codes were grouped in ever more categories until each group represented a coherent unit based on method of surgery, interventions performed, extent and site of the surgical procedure. Next two ID specialist (arbitrated by a third in case of disagreement) independently assigned country-specific codes to the resulting categories. Finally, specialist from each surgical discipline reviewed these assignments for their respective field.

**Results:**

A total number of 153 SALT (*Staphylococcus aureus* Surgical Site Infection Multinational Epidemiology in Europe) codes from 10 specialties were assigned to 15,432 surgical procedures. Almost 4000 (26%) procedure codes from the SALT coding system were classified as orthopaedic and trauma surgeries, thus this medical field represents the most diverse group within the SALT coding system, followed by abdominal surgical procedures with 2390 (15%) procedure codes.

**Conclusion:**

Mapping country-specific codes procedure codes onto to a limited number of coherent, internally and externally validated codes proofed feasible. The resultant SALT procedure code gives the opportunity to harmonize big data sets containing surgical procedures from international centres, and may simplify comparability of future international trial findings.

**Trial registration:**

The study was registered at clinicaltrials.gov under NCT03353532 on November 27^th^, 2017.

**Supplementary Information:**

The online version contains supplementary material available at 10.1186/s12874-022-01702-w.

## Background

The use of routine data will be essential in future healthcare research. Therefore, Harmonizing procedure codes is a first step to facilitate this approach as international research endeavour

One example is the investigation of surgical site infections (SSI). They are frequent hospital acquired complications [1–3] andprolong hospitalization, increase treatment costs, and are associated with poor outcome [4]. Ongoing clinical trials and surveillance programs evaluate the incidence of SSI worldwide on a national or regional basis in a limited number of procedures [1, 5–9]. Prior research demonstrated that surveillance of indicator operative procedures does not accurately reflect the overall burden of SSI. Thus, we initiated the SALT (*Staphylococcus aureus* Surgical Site Infection Multinational Epidemiology in Europe; NCT03353532) trial, a retrospective, multinational, multi-centre cohort study with a nested case–control part aiming to determine procedure specific *Staphylococcus aureus* SSI incidence for all surgical procedures in a sample of 15 centres (Supplement 1) from five European countries. The original SALT cohort included all adult undergoing surgery at these centres in 2016, excluding eye surgery, and compromised 178 902 patients – technical details have been reported before [7].

The inclusion of a sufficient number of patients to determine SSI incidence with meaningful precision for all common surgical procedures necessitated the use of routine data exported from hospital information system. While medical conditions are currently universally encoded using ICD-10, procedures are usually represented using country specific codes. Procedures codes are shaped by different health and reimbursement systems and thus tend to display a greater heterogeneity than would be expected for an exclusively medical coding system. To our knowledge currently no automated process allows the translations from one country specific procedure coding system to another or mapping into a universal system. One of the most extensive translation tables for Europe currently used is provided in the ECDC technical guidance document on SSI surveillance [10]. However, even this work provides translations for merely nine procedures in two systems (ICD-9 and NHSN).

We aimed to develop an international surgical procedure coding systems allowing for automatic translation and categorization of procedures documented in country-specific codes. This system aspires to be both comprehensive, i.e. encompassing all invasive surgical procedures, while providing a sufficient level of granularity suitable for epidemiological research. The presented system can easily be expanded to include both other coding systems and types of procedures (e.g. eye surgery).

## Methods

We developed a interantional procedure code including five European countries: France, Germany, Italy, Spain and the UK.

We utilized a multistep approach: First, all procedures codes and their associated plain-text procedure names were extracted from the SALT dataset. Next, plain-text procedures names were translated into English and procedures listed by similarity of name. In the following step, a team of physicians and data managers iteratively grouped procedures into increasingly smaller subsets until each group represented a coherent unit based on method of surgery (e.g. laparoscopic or open), interventions performed, extent and site of the surgical procedure. As a first validation step we determined group sizes resulting from assigning all patients from the original SALT cohort to these interim categories to avoid both to granular and to inhomogeneous groups. Next, external validity was discussed (i.e., if groups truly represented comparable procedures (e.g., left and right hemicolectomy vs open or laparoscopic excision of large intestine [V13; V14]). This step involved multiple iterations and adjustments until internal consensus was reached.

In the next step each resulting group and assigned procedures were again independently reviewed by two physicians. Whenever a consensus was found immediately, the next step was external review as described below. If suggestions deviated, a third contributor was involved before external review.

The resulting groupings were reviewed by external specialist surgeons from each of the surgical disciplines, except neurosurgery where no external specialist was available and thus an internal specialist was used, and board-certified infectious disease specialists. Surgeons (*n* = 8; RAM, MG, JAH, ML, COL, RR, GS, SW) from existing research-collaborations had been approached for this step previously. Reviewing surgeons were provided tables including both the original procedure name as well as the English translation.

**Fig. 1:**
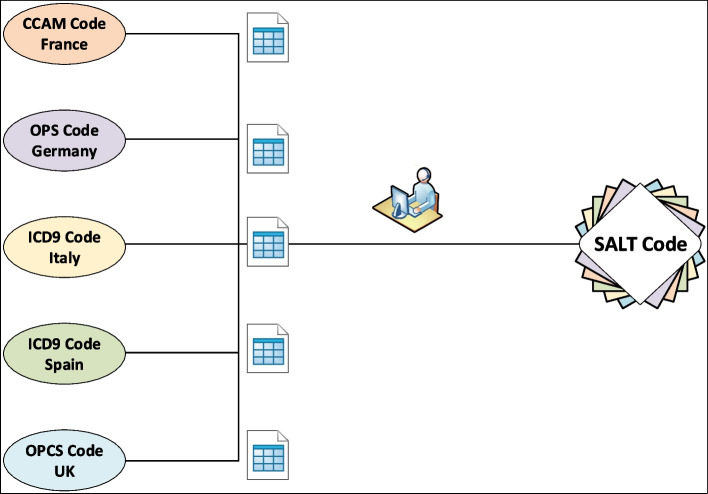
Composition of the SALT Code

Eye surgery and diagnostic procedures were excluded (Fig. 1 and Table 1).

**Table 1:** Inclusion and exclusion criteria for the salt coding system

Country	Code	To Include	To Exclude	Reason
Germany	OPS	Operations with 5-*		
			5–08 to 5–16	Eye surgery
			5–411	Bone marrow transplant
			5–890* and 5–91*	Superficial dermatologic interventions (tattoo, botulinum toxin injection, laser)
			5–93 to 5.98	Additional information, no surgical procedure
Spain/ Italy	ICD-9		00.01 to 00.96	"Other", not sufficiently specified procedure
			8.01 to 16.99	Eye surgery
			41.00 to 41.09	Bone marrow transplant
			87.01 to 99.99	Miscellaneous diagnostic and therapeutic procedures (incl. radiologic diagnostic)
			04.8; 04.81; 20.94; 34.92	Injection/Anaesthesia
			30.52	Chiropractic
			39.95 and 54.98	Dialysis
UK	OPCS		Chapter C	Eye surgery
			Chapter Y and Z	Subsidiary classification of methods of operations and sites of operation
			X41.1 and X.41.2	Insertion or removal of dialysis catheter
France	CCAM		Chapter 2	Eye surgery
			Chapter 5.3.2	Therapeutic interventions regarding the "blood" (incl. bone marrow transplant, transfusion)
			17.*	Miscellaneous diagnostic and therapeutic procedures (incl. radiologic diagnostic)
			18.*	Anaesthesia
			19.*	

* not further specified

*Abbreviations*: *CCAM* Classification commune des actes médicaux medical, engl. general classification of medical procedures, *ICD* International classification of diseases, *OPS* Operationen- und Prozedurenschlüssel, engl. operational and procedural keys, *OPCS* Office of Population Censuses and Surveys

After implementation of the SALT coding system and definition of surgical procedures, existing national ICD and OPS codes from France (CCAM v52), Germany (OPS-301), Italy (ICD-9-CM-2007, Italian version), Spain (CIE-9-MC-2014) and the UK (OPCS-4) were manually allocated to the SALT code based on the matching definitions of the codes facilitating cross-linking in between these coding systems. The SALT code database was built using Microsoft Access (Microsoft Corporation, Redmond, WA, USA). Entries were analysed for duplicates and editing/typing errors by applying various SQL (structured query language) queries.

## Results

The SALT coding system is based on a unique identifier code consisting of a three to four letter code indicating the medical field of the surgery (e.g., GYN for gynaecology) followed by a two-digit number for further classification (Table 2 and Supplementary Table S1).

**Table 2:** Overview of salt code with regard to surgical disciplines

Medical field of the surgery	Letter code	Digit code	N° of assigned procedure codes	[%] of assigned procedure codes
Dermatological surgery	DER	01 – 09	992	6
Ear, nose and throat surgery	ENT	01 – 10	1359	9
Gynaecological surgery	GYN	01 – 12	1118	7
Heart and cardiothoracic surgery	HCTH	01 – 13	1280	8
Neurosurgery	NSY	01 – 06	914	6
Oral and maxillofacial surgery	OMS	01 – 03	840	5
Orthopaedic and trauma surgery	OTS	01 – 29	3973	26
Urological surgery	URO	01 – 23	1208	8
Vascular surgery	VAS	01 – 11	1357	9
Visceral surgery	VIS	01 – 34	2390	15
(Premature termination of surgery)	(X)	(001)	(1)	0
**Total**	**11**	**153**	**15,432**	**100**

It facilitates a cross-linked, harmonized, and standardized encoding of surgical procedures from five major European countries: France, Germany, Italy, Spain and the UK. A total number of 15,432 surgical procedures are assigned to 153 SALT codes from 10 specialties (Table 2 and Table S1); 39% (*n* = 6025) and 34% (*n* = 5283) procedure codes within the SALT coding system are taken from the German OPS code and the French CCAM code, respectively (Table 3). The remaining 27% (*n* = 4124) procedure codes are derived from the Italian and Spanish version of ICD-9 (9% [*n* = 1316] and 12% [1816]) or the British OPCS (6% [*n* = 992]).

**Table 3:** Number of Codes included in the salt code, by surgical discipline

*Medical field* *(Letter Code)*	*N° Codes* *France*	*N° Codes Germany*	*N° Codes* *Italy*	*N° Codes Spain*	*N° Codes* *UK*	*N° Codes* *total*
DER	292	595	41	43	21	**992**
ENT	508	418	133	180	120	**1359**
GYN	289	375	156	200	98	**1118**
HCTH	491	434	106	185	64	**1280**
NSY	389	346	61	105	13	**914**
OMS	500	231	58	44	7	**840**
OTS	1150	1932	313	423	155	**3973**
URO	435	390	104	173	106	**1208**
VAS	565	489	95	107	101	**1357**
VIS	664	814	249	356	307	**2390**
(X)	0	1	0	0	0	**1**
**Total N°** **(Total %)**	**5283** **(34%)**	**6025** **(39%)**	**1316** **(9%)**	**1816** **(12%)**	**992** **(6%)**	***15,432***

Almost 4000 procedures codes (26%) from the SALT coding system are classified as orthopaedic and trauma surgeries, thus this medical field represents the most diverse group within the SALT coding system, followed by visceral surgical procedures (2390 procedure codes, 15%). “Operations on bone” (OTS01, 1071 procedures) and “Other Operations on muscles, tendons, fascia and bursa” (OTS24, 565 procedures) are the SALT codes with the highest number of procedure codes assigned to. Further details are depicted in Supplementary Table S1.

## Discussion

To facilitate the conduct of ongoing clinical trials analysing incidence and impact of SSI internationally, we developed a coding system harmonizing country-specific procedure codes used in selected European countries.

Surveillance by hospital infection prevention programs often depends on unproven screening strategies to identify patients with possible SSI such as screening of readmissions, review of daily microbiology results, and surgeon self-report. While clinical scoring systems have been validated for the detection of SSI [11, 12], these scores are not routinely documented in EHRs and not readily calculated retrospectively from available data. While a specialised coding system is important for accounting purposes in each country, an international coding system is needed to harmonize data within large clinical trials and facilitate the inclusion of routine clinical and administrate data. The use of an international procedure code has the potential to improve SSI detection and surveillance and allows for more standardized inter-hospital comparison on an international level.

Using the presented coding systems future prospective trials, retrospective analyses and routine surveillance efforts can streamline international collaboration by obviating the need for manual translation of surgical procedure codes. While our coding system was developed in the context of SSI research, it can be utilized in all international trails comparing aspects of surgery, e.g., indications, outcomes, costs, etc. Future efforts could also expand to code both horizontally, i.e., by mapping other country specific codes onto the SALT code, or vertically, i.e., by adding new codes. Where needed, granularity could be added with minimal modifications by expanding the namespace while preserving compatibility (e.g., VCH1-A, VCH1-B, etc.).

The international comparison of social and health systems is constantly growing in significance as emphasized by the currently ongoing SARS-CoV-2 pandemic. National as well as international harmonization efforts comprise computer modelling and decision support systems to guide evidence-informed medicine. However, to be effective, mutual coding not only of procedures, but also of further tasks from a whole system’s perspective including health, social, housing, employment, education, and justice, need to be targeted. Novel approaches including those aspects aim to create harmonized codes by automated computerized techniques [13, 14]. Automated code harmonization and machine learning models, currently being validated, may also be subject of future trials in the context of surgical procedures.

Our approach has several limitations. As it has been created manually, the implementation of in- and exclusion criteria is error-prone: National codes contain several procedures which are clearly not surgeries, e.g., haemodialysis. We believe that our iterative approach with multiple, independent round of validation by both ID physician and surgeons minimizes to potential for human error. In addition, the basis for our codes was an export of surgical procedures performed procedures at participating centres. The code may thus not be universal neither complete. However, based on the sheer number of included patients (> 170.000) we believe to have covered all quantitatively relevant procedures. Furthermore, this code excludes eye surgery, as well as paediatric surgery and is limited to France, Germany, Italy, Spain and the UK.

The current edition of SALT and future updated editions will be accessible online in a machine-readable format. Further editions of the here proposed coding system would need to include the complete spectrum of codes in all countries as well as to include further countries. It may not only be utilized for trials within the frame of infectious disease and infection control, but also for a larger scope of scientific research questions in various medical fields.

## Conclusion

This Europe-wide procedure code gives the opportunity to harmonize big data sets containing surgical procedures from international centres. If it is utilized, adapted and expanded in future research, we encourage research to share the resulting codes and offer to publish them along the original coding table.

## Supplementary Information

**Additional file 1.**

## Data Availability

Data and material will be available upon request to the corresponding author (Dr. Sibylle C. Mellinghoff, University Hospital of Cologne; sibylle.mellinghoff@uk-koeln.de). In addition, data will be available at the Kölner UniversitätsPublikationsServer (KUPS).

## Abbreviations

CCAM: Classification commune des actes médicaux medical, engl. general classification of medical procedures
eCDC: European Centre for Disease Prevention
ICD: International classification of diseases
NHSN: National Healthcare Safety Network
OPCS: Office of Population Censuses and Surveys
OPS: Operationen- und Prozedurenschlüssel, engl. operational and procedural keys
SA: Staphylococcus aureus
SSI: Surgical site infections
