# Supplementary material for: Harmonized procedure coding system for surgical procedures and analysis of surgical site infections (SSI) of five European countries
Source: BMC Med Res Methodol. 2022 Aug 12;22:225. doi: 10.1186/s12874-022-01702-w (PMC9374282; doi:10.1186/s12874-022-01702-w)
Supplement: Supplementary file 1 [file 12874_2022_1702_MOESM1_ESM.docx]

Table S1. SALT Code with all Included Codes

| SALT Code | Description | N° of Assigned Codes | [%] of Assigned Codes |
| --- | --- | --- | --- |
| DER01 | Incision and excision of skin and subcutaneous tissue | 351 | 2.3 |
| DER02 | Wound Debridement | 120 | 0.8 |
| DER03 | Excision and reconstruction of sinus pilonidalis: Excision | 8 | 0.1 |
| DER04 | Excision of nail bed | 22 | 0.1 |
| DER05 | Autograft of skin to head or neck | 100 | 0.6 |
| DER06 | Local flap of skin and subcutaneous tissue | 144 | 0.9 |
| DER07 | Facial plastic surgery | 146 | 0.9 |
| DER08 | Temporary soft tissue coverage | 30 | 0.2 |
| DER09 | Other burn or chemical burn operations | 71 | 0.5 |
| ENT01 | Surgery on middle ear and inner ear | 293 | 1.9 |
| ENT02 | Operations on mastoid | 23 | 0.1 |
| ENT03 | Operations on the nose | 204 | 1.3 |
| ENT04 | Operations on the nasal sinuses | 124 | 0.8 |
| ENT05 | Operations on the tongue | 62 | 0.4 |
| ENT06 | Operations on the salivary gland and salivary duct | 100 | 0.6 |
| ENT07 | Operations at the mouth | 252 | 1.6 |
| ENT08 | Operations on the pharynx | 132 | 0.9 |
| ENT09 | Tonsillectomy | 42 | 0.3 |
| ENT10 | Operations on the larynx | 127 | 0.8 |
| GYN01 | Surgery on ovary and fallopian tubes | 125 | 0.8 |
| GYN02 | Open surgery of ovary and fallopian tubes | 94 | 0.6 |
| GYN03 | Surgery on uterus and cervix uteri | 187 | 1.2 |
| GYN04 | Open surgery on uterus and cervix uteri | 114 | 0.7 |
| GYN05 | Surgery on vagina and recto-uterine pouch | 168 | 1.1 |
| GYN06 | Surgery on vulva | 86 | 0.6 |
| GYN07 | Vaginal delivery | 34 | 0.2 |
| GYN08 | Caesarean section | 35 | 0.2 |
| GYN09 | Other obstetric operations | 66 | 0.4 |
| GYN10 | Breast excision and resection | 122 | 0.8 |
| GYN11 | Plastic surgery of breast augmentation and reduction | 55 | 0.4 |
| GYN12 | Breast surgery | 32 | 0.2 |
| HCTH01 | Operations on the trachea | 94 | 0.6 |
| HCTH02 | Operations of a bronchus | 6 | 0.0 |
| HCTH03 | Open surgery of the lung and pleura (reconstruction and removal) | 212 | 1.4 |
| HCTH04 | Minimally invasive surgery of the lung and pleura (reconstruction and removal) | 70 | 0.5 |
| HCTH05 | Lung transplantation | 16 | 0.1 |
| HCTH06 | Open surgery of the mediastinum | 47 | 0.3 |
| HCTH07 | Minimally invasive surgery of the Mediastinum (reconstruction and removal) | 9 | 0.1 |
| HCTH08 | Operation on the diaphragm | 33 | 0.2 |
| HCTH09 | Other operations on the chest wall | 52 | 0.3 |
| HCTH10 | Atrial septum and valve repair surgery | 258 | 1.7 |
| HCTH11 | Minimally invasive atrial septum and valve repair surgery | 24 | 0.2 |
| HCTH12 | Revascularization of the heart | 112 | 0.7 |
| HCTH13 | Other operations on the heart and pericard | 309 | 2.0 |
| HCTH14 | Heart and heart-lung replacement by transplantation or ECMO | 38 | 0.2 |
| NSY01 | Operations on scull, brain and meninges | 419 | 2.7 |
| NSY02 | Access to the vertebral column, extradural | 59 | 0.4 |
| NSY03 | Operations on spinal cord and spinal cord structures | 121 | 0.8 |
| NSY04 | Reconstruction of a nerve and nerve plexus | 261 | 1.7 |
| NSY05 | Insertions of neurostimulator adjacent to spinal cord | 41 | 0.3 |
| NSY06 | Stereotactic operations on the skull, brain and meninges | 13 | 0.1 |
| OMS01 | Operations of scull fractures | 162 | 1.0 |
| OMS02 | Operations on temporomandibular joint and bones of scull | 243 | 1.6 |
| OMS03 | Operations on teeth, gum and alveoli | 435 | 2.8 |
| OTS01 | Operations on bone | 1071 | 6.9 |
| OTS02 | Open reduction and internal fixation of a fracture or slipped epiphysis | 208 | 1.3 |
| OTS03 | Open reduction of a fractured long tubular bone | 278 | 1.8 |
| OTS04 | Primary open reduction of fracture of short bone | 95 | 0.6 |
| OTS05 | Open reduction of a pelvic fracture | 20 | 0.1 |
| OTS06 | Reduction of a joint dislocation | 125 | 0.8 |
| OTS07 | Open surgery of a joint | 291 | 1.9 |
| OTS08 | Open surgery on the knee | 96 | 0.6 |
| OTS09 | Arthroscopic joint surgery | 156 | 1.0 |
| OTS10 | Arthroscopic surgery of the knee | 57 | 0.4 |
| OTS11 | Arthroscopic surgery of the shoulder | 27 | 0.2 |
| OTS12 | Primary total prosthetic replacement of hip joint | 38 | 0.2 |
| OTS13 | Revision, replacement and removal of total prosthetic hip joint | 74 | 0.5 |
| OTS14 | Total prosthetic replacement of knee joint | 25 | 0.2 |
| OTS15 | Revision, replacement and removal of total prosthetic knee joint | 40 | 0.3 |
| OTS16 | Primary total prosthetic replacement of a joint of the upper limb | 29 | 0.2 |
| OTS17 | Revision, replacement and removal of prosthetic joint of the upper limb | 48 | 0.3 |
| OTS18 | Primary total prosthetic replacement of a joint of the foot | 5 | 0.0 |
| OTS19 | Revision, replacement and removal of a prosthetic joint of the foot | 5 | 0.0 |
| OTS20 | Other joint plastic procedures | 42 | 0.3 |
| OTS21 | Operations on the vertebral column | 239 | 1.5 |
| OTS21a | Osteosynthesis of the vertebral column | 24 | 0.2 |
| OTS22 | Spondylodesis | 66 | 0.4 |
| OTS23 | Operations on tendons and joints of the hand | 189 | 1.2 |
| OTS24 | Other Operations on muscles, tendons, fascia and bursa | 565 | 3.7 |
| OTS25 | Reconstruction and re-amputation of upper limb | 26 | 0.2 |
| OTS26 | Amputation and disarticulation of upper limb | 40 | 0.3 |
| OTS27 | Amputation and disarticulation of lower limb | 56 | 0.4 |
| OTS28 | Reconstruction and revision of amputation stump | 14 | 0.1 |
| OTS29 | Operations on soft tissue | 24 | 0.2 |
| URO01 | Open surgery on the kidney | 108 | 0.7 |
| URO02 | Minimally invasive operations on the kidney | 31 | 0.2 |
| URO03 | Open nephrectomy | 71 | 0.5 |
| URO04 | Minimally invasive nephrectomy | 10 | 0.1 |
| URO05 | Open surgery on ureter | 82 | 0.5 |
| URO06 | Minimally invasive operations on ureter | 85 | 0.6 |
| URO07 | Urinary diversion surgery | 48 | 0.3 |
| URO08 | Removal of stones, foreign bodies and tamponades of bladder | 10 | 0.1 |
| URO09 | Open surgery on the bladder | 93 | 0.6 |
| URO10 | Minimally invasive operations on the bladder | 43 | 0.3 |
| URO11 | Open surgery on the urethra | 120 | 0.8 |
| URO12 | Minimally invasive operations on the urethra | 31 | 0.2 |
| URO13 | Other operations on retroperitoneum and urinary tract | 101 | 0.7 |
| URO14 | Open surgery on the prostate | 26 | 0.2 |
| URO15 | Minimally invasive operations on the prostate | 37 | 0.2 |
| URO16 | Open prostatectomy | 23 | 0.1 |
| URO17 | Minimally invasive prostatectomy | 4 | 0.0 |
| URO18 | Operations on scrotum | 38 | 0.2 |
| URO19 | Operations on testis | 59 | 0.4 |
| URO20 | Orchidectomy | 23 | 0.1 |
| URO21 | Operations on Funiculus spermaticus, Epididymis and Ductus deferens | 21 | 0.1 |
| URO22 | Operations on penis | 121 | 0.8 |
| URO23 | Transplantation of kidney | 23 | 0.1 |
| VAS01 | Open Embolectomy, Thrombectomy und Endarterectomy of blood vessels | 153 | 1.0 |
| VAS02 | Operations on blood vessels by replacement or re-anastomosis | 251 | 1.6 |
| VAS03 | Excision of blood vessels and extraction of transplant | 39 | 0.3 |
| VAS04 | Stripping of varicose veins | 17 | 0.1 |
| VAS05 | Ligation of blood vessels | 138 | 0.9 |
| VAS06 | Endovascular insertion of stent graft | 82 | 0.5 |
| VAS07 | Open insertion of stent grafts | 30 | 0.2 |
| VAS08 | Angioplasty | 28 | 0.2 |
| VAS09 | Insertion of arteriovenous prothesis | 109 | 0.7 |
| VAS10 | Other surgical procedures on blood vessels | 185 | 1.2 |
| VAS11 | Surgery on lymph nodes and vessels | 325 | 2.1 |
| VIS01 | Operations on thyroid gland and parathyroid gland | 90 | 0.6 |
| VIS02 | Operations on adrenal gland | 36 | 0.2 |
| VIS03 | Operations on hypophysis | 20 | 0.1 |
| VIS04 | Thymectomy | 17 | 0.1 |
| VIS05 | Operations on bone marrow | 16 | 0.1 |
| VIS06 | Splenectomy | 20 | 0.1 |
| VIS07 | Minimally invasive surgery on the oesophagus | 82 | 0.5 |
| VIS08 | Open surgery on the stomach | 310 | 2.0 |
| VIS09 | Minimally invasive surgery on the stomach | 70 | 0.5 |
| VIS10 | Other operations on the intestine | 190 | 1.2 |
| VIS11 | Open local excision of small intestine | 40 | 0.3 |
| VIS12 | Laparoscopic local excision of small intestine | 32 | 0.2 |
| VIS13 | Open local excision of large intestine | 12 | 0.1 |
| VIS14 | Laparoscopic local excision of large intestine | 27 | 0.2 |
| VIS15 | Open resection of small intestine | 26 | 0.2 |
| VIS16 | Open resection of large intestine | 122 | 0.8 |
| VIS17 | Minimally invasive resection of large intestine | 39 | 0.3 |
| VIS18 | Creation of enterostomy | 77 | 0.5 |
| VIS19 | Open appendectomy | 28 | 0.2 |
| VIS20 | Laparoscopic appendectomy | 5 | 0.0 |
| VIS21 | Open surgery on rectum | 82 | 0.5 |
| VIS22 | Minimally invasive surgery on rectum | 67 | 0.4 |
| VIS23 | Anal and perianal surgery | 174 | 1.1 |
| VIS24 | Hepatectomy | 91 | 0.6 |
| VIS25 | Transplantation of liver | 6 | 0.0 |
| VIS26 | Operations on the liver | 40 | 0.3 |
| VIS27 | Open cholecystectomy/ cholecystostomy | 26 | 0.2 |
| VIS28 | Minimally invasive cholecystectomy/ cholecystostomy | 19 | 0.1 |
| VIS29 | Open surgery on the bile duct | 19 | 0.1 |
| VIS30 | Minimally invasive surgery on the bile duct | 89 | 0.6 |
| VIS31 | Open excision and resection of pancreas | 96 | 0.6 |
| VIS32 | Laparoscopic surgery on pancreas | 30 | 0.2 |
| VIS33 | Repair of inguinal hernia | 207 | 1.3 |
| VIS34 | Operations on the abdominal wall | 185 | 1.2 |
| X001 | Premature termination of surgery | 1 | 0.0 |

Supplement 1 – participating centres by country

France (Centre Hospitaler Universitaire de Limoges, Centre Hospitalier Régional Universitaire de Tours and Centre Hospitalier Départemental Vendée), Germany (Universitätsklinikum Bonn, Universitätsklinikum Cologne, Universitätsklinikum Jena and Universitätsklinikum Ludwig-Maximilians-Universität [LMU]-Munich), Italy (Azienda Sanitaria Universitaria Udine), United Kingdom (NHS Manchester), Spain (Institut Hospital del Mar d’Investigacions Mèdiques [IMIM] de Barcelona, Hospital Clínic de Barcelona, Hospital General Universitario Gregorio Marañón Madrid, Hospital Universitario Ramón y Cajal Madrid and Hospital Universitari I Politècnic La Fe Valencia)
